# Comparative Biomechanical and Microstructural Analysis of Native versus Peracetic Acid-Ethanol Treated Cancellous Bone Graft

**DOI:** 10.1155/2014/784702

**Published:** 2014-02-11

**Authors:** Juliane Rauh, Florian Despang, Jorgen Baas, Cornelia Liebers, Axel Pruss, Michael Gelinsky, Klaus-Peter Günther, Maik Stiehler

**Affiliations:** ^1^University Centre for Orthopaedics and Traumatology, University Hospital Carl Gustav Carus, Technische Universität Dresden, 01307 Dresden, Germany; ^2^Centre for Translational Bone, Joint and Soft Tissue Research, University Hospital and Medical Faculty Carl Gustav Carus, Technische Universität Dresden, 01307 Dresden, Germany; ^3^Orthopaedic Research Laboratory, Aarhus University Hospital, 8000 Aarhus, Denmark; ^4^Institute of Transfusion Medicine, Tissue Bank, Charité-Universitätsmedizin Berlin, 10117 Berlin, Germany

## Abstract

Bone transplantation is frequently used for the treatment of large osseous defects. The availability of autologous bone grafts as the current biological gold standard is limited and there is a risk of donor site morbidity. Allogenic bone grafts are an appealing alternative, but disinfection should be considered to reduce transmission of infection disorders. Peracetic acid-ethanol (PE) treatment has been proven reliable and effective for disinfection of human bone allografts. The purpose of this study was to evaluate the effects of PE treatment on the biomechanical properties and microstructure of cancellous bone grafts (CBG). Forty-eight human CBG cylinders were either treated by PE or frozen at −20°C and subjected to compression testing and histological and scanning electron microscopy (SEM) analysis. The levels of compressive strength, stiffness (Young's modulus), and fracture energy were significantly decreased upon PE treatment by 54%, 59%, and 36%, respectively. Furthermore, PE-treated CBG demonstrated a 42% increase in ultimate strain. SEM revealed a modified microstructure of CBG with an exposed collagen fiber network after PE treatment. We conclude that the observed reduced compressive strength and reduced stiffness may be beneficial during tissue remodeling thereby explaining the excellent clinical performance of PE-treated CBG.

## 1. Introduction

Bone grafting as one of the most common orthopedic procedures is frequently used for the treatment of osseous defects due to trauma, tumor, degenerative or congenital disorders, and infection as well as to periprosthetic bone loss [1]. Autografting is currently regarded as the gold standard providing a vital, osteoinductive, vascularized, and three-dimensional structural construct for the support of localized bone regeneration [2, 3]. However, autologous bone grafting is associated with postoperative donor site morbidity, for example, neurovascular injury, persisting pain, hematoma, and fracture as well as limited availability and reduced quality in old patients and patients with bone metabolism compromising comorbidities, for example, osteoporosis [4, 5]. In this context, the application of bone allografts possessing both osteoconductive and at best partially osteoinductive properties, usually provided by local bone or tissue banks, denotes an appealing alternative [6, 7].

In order to minimize the potential risk of transmission of infectious agents, for example, human immunodeficiency virus [8], hepatitis viruses [9], or bacteria [10], safe disinfection methods of bone tissue transplants are of utmost importance for clinical use [6, 8]. Currently, several disinfection methods are available for the generation of transplantable bone allografts, for example, chemical disinfection [11, 12], thermal treatment [13, 14], treatment with supercritical CO_2_ [15], and beta or gamma irradiation [16, 17]. Additionally, high hydrostatic pressure treatment of allografts is available [18]. While some disinfection methods, for example, gamma irradiation or treatment with chemical agents, inactivate pathogens very efficiently but influence the biomechanical or osteoinductive properties of bone grafts negatively [13, 18–24] alternative methods, for example, using high hydrostatic pressure or thermal treatment with 82.5°C, showed only a limited effect on the viability of pathogens while preserving the biomechanical integrity [13, 18]. Among the chemical disinfection procedures the peracetic acid-ethanol (PE) treatment has been proven an effective method for human CBG and has been previously validated extensively for the elimination of relevant viruses, bacteria, fungi, and even spores [25, 26]. Notably, the advantage of being sporicidal in contrast to other available techniques makes PE treatment a reliable method for the disinfection of tissue transplants. Recently, our group demonstrated the successful vitalization of PE-treated CBA with mesenchymal stromal cells under good manufacturing practice conditions [27].

Haimi et al. found that PE treatment did not influence the biomechanical properties of *cortical* bone grafts significantly using a three-point bending test [28]. The aim of this study was to investigate the effects of PE disinfection on the mechanical properties of *cancellous* bone transplants which, to the authors' best knowledge, has not been performed previously.

## 2. Materials and Methods 

### 2.1. Bone Specimen

CBG samples with cylindrical geometry (diameter ~12 mm, length ~15 mm) were processed from symmetric locations of proximal tibiae from four cadavers (3 males, 22/44/55 years, and one female, 43 years). The use of CBG for research purposes has been approved by the Ethics Committee of the Charité-Universitätsmedizin Berlin (EK/CHB/13062003). Dimensions, weight, and density of the samples are summarized in Table 1. A total of *N* = 48 CBG samples (6 per donor and 24 per group) were used for the study.

Twenty-four native cancellous bone (NCB) samples were frozen immediately after harvest and maintained at −20°C until use. The remaining *n* = 24 specimens underwent PE treatment as previously described by one of us [25]. Briefly, after fat and connective tissue were removed the CBG samples were rinsed under high pressure with sterile water at 37°C for 30 min. Remaining fat was removed by incubation in a mixture of chloroform (99.4%) and methanol (99.8%) v/v, 2/1 under constant agitation for 2 h. To remove chloroform the samples were sonicated eight times in methanol for 15 min. Methanol was removed by flushing the tissue twice with sterile deionized water. Disinfection was done under constant agitation at low pressure (200 mbar) and room temperature (RT) for 4 h. The CBA samples were covered with v/v 1/7.5 peracetic acid solution (i.e., 2% peracetic acid, 96% ethanol, aqua ad iniectabilia (ratio v/v/v 2/1/1)) and consequently freeze-dried, packed sterile, and kept at RT for later use. PE-treated CBG has a conferred admission for medicinal drugs (according to Section 21, 105, German Medicines Law (AMG)).

### 2.2. Biomechanical Test

**Figure 1:**
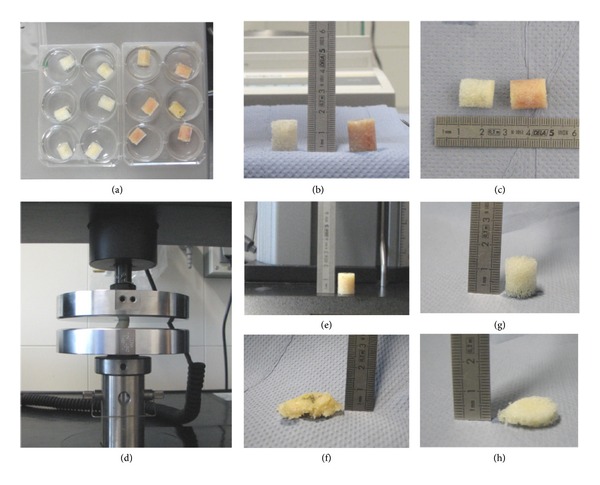
(a)–(h) Biomechanical testing. Native cancellous bone (NCB) (right) and peracetic acid-ethanol (PE)-treated (left) cancellous bone allograft (CBG) cylinders were incubated for 24 h in saline prior to biomechanical testing (a). The dimensions of the cylindric bone allografts (12 × 15 mm) are shown in (b) and (c). Axial compression was performed using an Instron Wolpert 5566 material testing device (d). NCB (e, f) and PE-treated (g), (h) CBG cylinders before (e), (g) and after (f), (h) axial compression loading.

Testing was performed on paired specimens from the same donor and equivalent donor sites, where each specimen within a pair was randomly allocated to treatment or control group. Twenty-four hours prior to the biomechanical testing the specimens were transferred to a physiological saline solution and incubated at RT (Figure 1(a)). The apparent densities of NCB and PE-treated specimens were calculated by determining the weight divided by the total volume [29]. The wet weight was measured with a balance (Satorius BP221S, Göttingen, Germany), and the total sample volume was determined by using an electronic micrometric caliper (PMS 150, 0–150 mm, Hitachi, Tokyo, Japan).

Mechanical testing was performed at RT using an Instron 5566 material testing device; Merlin Software was used for data acquisition (Instron Wolpert, Darmstadt, Germany). For axial compression each specimen was placed between two platens and compressed until failure with maximum load of 10 kN at a constant velocity of 0.07 mm/s (Figure 1(d)). Compressive strength, Young's modulus, ultimate strain, and fracture energy were calculated from stress-strain curves based on force-displacement data using the following equations:

(1) $$\begin{array}{c} \text{Compressive}\;\;\text{strength}\;\left(\sigma \;\text{in}\;\text{MPa}\right):\sigma =\frac{\text{force}\;\left(F\right)}{\pi *r^{2}} \end{array}$$

with area perpendicular to force axis.

Young's modulus was calculated from the linear slope of the stress-strain curve:

(2) Young's  modulus (E  in  MPa):E=ΔσΔεwith  strain  ε=Δ length  original  length.

Ultimate strain characterizes the ability of deformation and was calculated by the alteration of length relating to the original length after failure:

(3) $$\begin{array}{c} \text{Ultimate}\;\;\text{strain}\;\left(\varepsilon \;\text{in}\;\%\right):\varepsilon =\frac{\Delta \;\text{length}}{\text{original}\;\text{length}}*100\%. \end{array}$$

Fracture energy was calculated as the area enclosed by the stress-strain curve until the point of failure by means of Origin software (OriginLab, Northhampton, MA, USA):

(4) $$\begin{array}{c} \text{Fracture}\;\;\text{energy}\;\;\left(\text{FE}\;\;\text{in}\;\;\text{N}\;\text{mm}\right):\text{FE}=\overset{\text{failure}}{\underset{0}{\int }}F\left(x\right)*dx. \end{array}$$

### 2.3. Histology

Frozen NCB and PE-treated specimens were fixed in 4% neutral buffered formaldehyde (SAV Liquid Production, Flintsbach a. Inn, Germany) for 24 h at 4°C. After decalcification overnight in EDTA (Osteosoft, Merck, Darmstadt, Germany) samples were dehydrated in increasing ethanol concentrations and xylene at RT for 11 h. Specimens were embedded in paraffin (Thermo Fisher Scientific GmbH, Dreieich, Germany), and cross sections of 10 *μ*m thickness were obtained from the cured specimen blocks using a Leica RM2055 microtome (Leica, Nussloch, Germany).

Slices were deparaffined with xylene, incubated in decreasing ethanol concentrations and rehydrated with distilled water. Staining was performed with hematoxylin and eosin (H&E), Giemsa's stain (Merck, Darmstadt, Germany) and Alizarin 13203 (MORPHISTO GmbH Frankfurt am Main, Germany). Pictures were taken with a digital camera AxioCam MRc on an ApoTome Zeiss Imager Z1 using AxioVision software (Carl Zeiss MicroImaging GmbH, Jena, Germany).

### 2.4. Scanning Electron Microscopy (SEM)

NCB and PE-treated CBG samples were fixed for 24 h at 4°C in 2% glutaraldehyde solution (Sigma-Aldrich Chemie GmbH, Steinheim, Germany) followed by dehydration with increasing ethanol series (7 steps for 10 min each), critical point drying using a CPD 030 Critical Point Dryer (BAL-TEC GmbH, Schalksmühle, Germany) and final carbon coating with a sputtering unit EM CED030 (Leica, Wetzlar, Germany).

The microstructure of specimens was imaged by digital scanning electron microscope Zeiss DSM 982 Gemini (Carl Zeiss, Oberkochen, Germany) equipped with a field emission gun. The microscope was operated with an acceleration voltage of 5 kV and a working distance of 6 mm. To optimize data visualization, the acquired images were subsequently brightness and contrast adjusted using Photoshop Image Editing Software (Adobe Systems GmbH, Munich, Germany).

### 2.5. Statistics

Statistical data analysis was performed using GraphPad Prism 5.0 software (La Jolla, CA, USA) using a two-sided Mann-Whitney test and statistical significance considered if *P* < 0.05. Data is presented as mean ± standard error of the mean (SEM).

## 3. Results 

### 3.1. Biomechanical Testing

**Figure 2:**
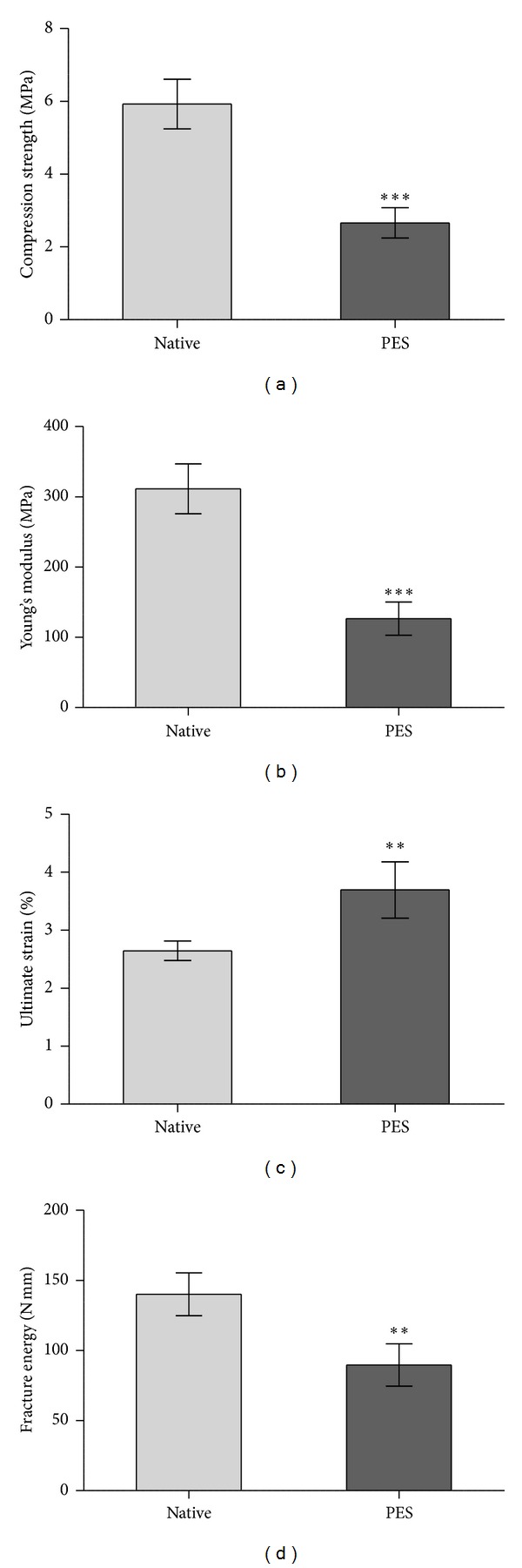
(a)–(d) Biomechanical data of native cancellous bone (NCB) and PE-treated cancellous bone allograft cylinders after axial compression testing. Compression strength (a), Young's modulus (b), ultimate strain (c) and fracture energy (d) are represented as mean ± standard error of the mean (*n* = 24; 4 donors with *n* = 6 per donor; *** = *P* < 0.0001, ** = *P* < 0.01, Mann-Whitney test).

Compression strength levels of NCB and PE-treated samples were 5.9 ± 0.7 MPa and 2.7 ± 0.4 MPa, respectively, corresponding to a 54% reduction in compressive strength by PE treatment (*P* < 0.0001, Figure 2(a)). The elastic properties as quantified by Young's modulus of NCB and PE-treated samples were 311.4 ± 34.7 MPa and 126.5 ± 23.6 MPa, respectively (*P* < 0.0001, Figure 2(b)), denoting a 59% reduction in stiffness by PE treatment. The degree of ultimate strain was significantly increased by PE treatment by 42% (2.6 ± 0.2% versus 3.7 ± 0.5%, *P* < 0.001, Figure 2(c)). Fracture energy levels of NCB and PE-treated specimens were 140.1 ± 15.3 N mm and 89.7 ± 15.1 N mm, respectively, corresponding to a 36% reduction by PE treatment (*P* < 0.001, Figure 2(d)).

**Figure 3:**
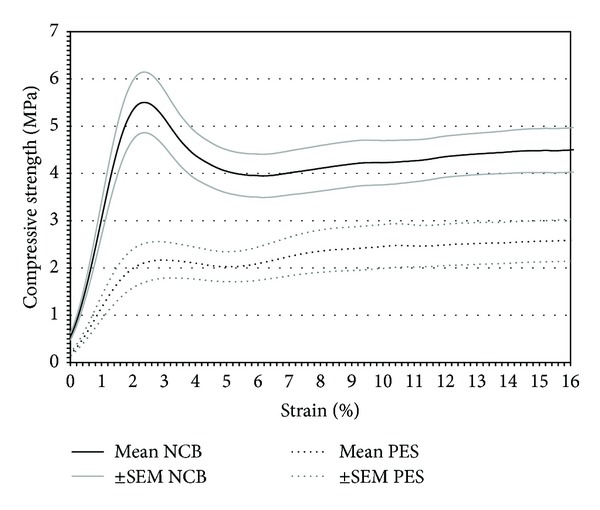
Stress-strain diagram of native cancellous bone (NCB) and peracetic acid-ethanol (PE)-treated cancellous bone allograft (CBG) cylinders. The black line represents the mean values of NCB; gray lines show the standard error of the mean. The dotted black line corresponds to the mean value of the PE-treated CBG; dotted gray lines show the standard error of the mean.

**Figure 4:**
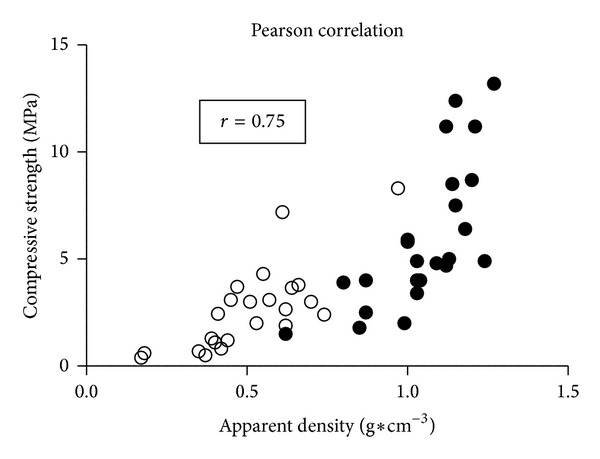
Correlation of compression strength and density values of native cancellous bone (NCB) and peracetic acid-ethanol (PE)-treated cancellous bone allograft (CBG) cylinders. Each point corresponds to the measurement of one specimen. Open symbols correspond to PE-treated CBG and filled symbols to NCB specimen. The correlation between the two data sets is significant with *r* = 0.75 and *P* < 0.0001.

After compression testing a slight stretching of PE-treated CBG but not NCB specimen demonstrated maintained elastic properties as observed by a gain in height by approximately 1 mm to 3 mm as displayed in Figure 1(h). The NCB samples, on the other hand, remained completely in the compressed state (Figure 1(f)).  Table 2 summarizes the donor-dependent data of compressive strength, Young's modulus, ultimate strain, and fracture energy. Specimens of donor 2 (male, 22 years) showed the strongest intergroup differences regarding compressive strength, Young's modulus, and ultimate strain. None of the investigated parameters of the PE-treated versus the NCB specimen from donor 3 (male, 44 years) were significantly different (Table 2). Donor 1 (male, 50 years) demonstrated the strongest intergroup differences in fracture energy with 61% reduction compared to the untreated CBG. Approximately 40% reduction in compressive strength, Young's modulus and fracture energy were detected for donor 4 (female, 43 years).  Figure 3 depicts a summarizing stress-strain diagram of the averaged values of the groupwise compression data highlighting intergroup changes in biomechanical behavior. In addition, the differences of elastic moduli are corresponding to slope steepness of the stress-strain curve. The apparent density (Table 1) of the CBG specimens was reduced by 51% by PE treatment.  Figure 4 shows the influence of apparent density on the compressive strength of NCB and PE-treated specimens. We observed a strong positive correlation between compressive strength and apparent density of the specimen (*P* < 0.0001; *r* = 0.75).

### 3.2. Histological Analysis

**Figure 5:**
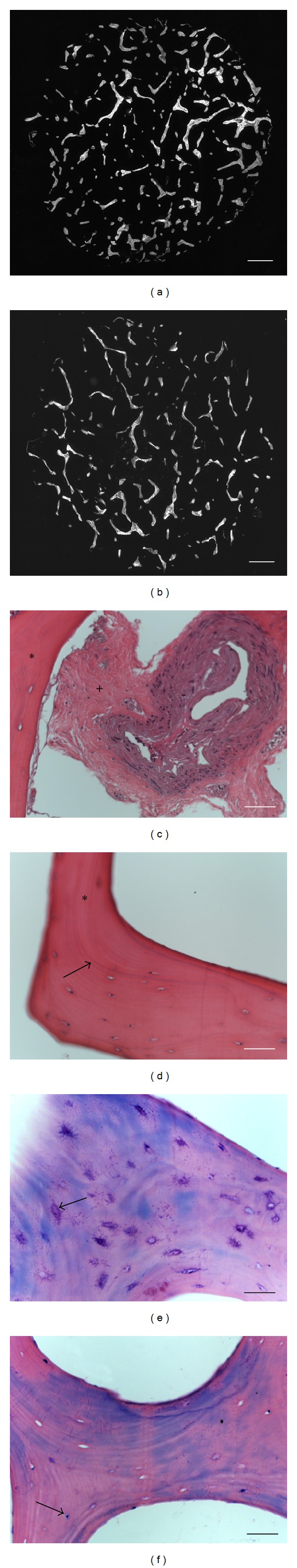
(a)–(f) Histological analysis of native cancellous bone (NCB) and peracetic acid-ethanol (PE)-treated cancellous bone allograft (CBG). Fluorescence microscopic image of cross sections from NCB (a) and PE-treated CBG (b) after Alizarin staining (scale bar = 1000 *μ*m). Micrographs of NCB (c) and PE-treated CBG (d) after hematoxylin and eosin staining demonstrating connective tissue in the NCB sample (scale bar = 50 *μ*m). Asterisks and plus sign denote cancellous bone and connective tissue, respectively. Arrow indicates crescent-shaped lamellae running parallel to the bone marrow interface; no tissue is visible in PE-treated CBG (d). Histologic sections of NCB (e) and PE-treated CBG (f) after Giemsa staining (scale bar = 50 *μ*m). Arrows correspond to osteocytes colored in dark purple. The fine cellular structures of osteocytes with dendritic processes and nuclei of NCB are clearly visible. In contrast, sections of PE-treated CBG demonstrate pores from bone forming osteocytes with their rested nuclei with remnant cellular structures after PE treatment.

Histological overview cross sections of NCB (Figure 5(a)) and PE-treated CBG (Figure 5(b)) showed no apparent differences in trabecula size and shape. Micrographs of HE stained sections revealed connective tissue in NCB samples (Figure 5(c)) whereas no tissue was found in the pores of PE-treated CBG section due to disinfection process (Figure 5(d)). Fine cellular structures of osteocytes with dendritic processes and nucleus are visible in NCB samples (Figure 5(e)). In contrast, sections of PE-treated CBG only demonstrated the remaining pores of bone forming osteocytes with their rested nucleus (Figure 5(f)).

### 3.3. Scanning Electron Microscopy Analysis

**Figure 6:**
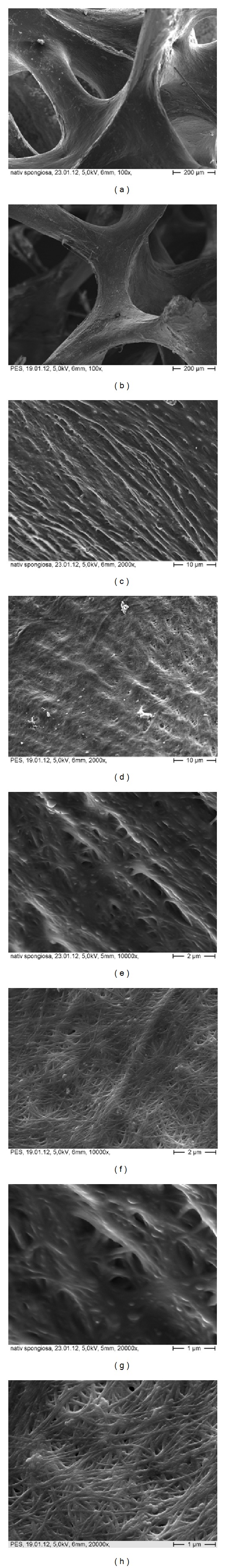
(a)–(h) Representative SEM images of native cancellous bone (NCB) and peracetic acid-ethanol (PE)-treated cancellous bone allograft (CBG) demonstrating untreated NCB ((a), (c), (e), and (g)) and PE-treated CBG ((b), (d), (f), and (h)) with collagen fibers closely associated with mineral phase. A more exposed biopolymer network is visible after PE treatment ((f), (h)). Micrographs were acquired at 100x ((a), (b)), 2 000x ((c), (d)), 10 000x ((e), (f)), and 20 000x (g, h) magnification.

Figure 6 depicts two parallel sets of SEM micrographs from lower to higher magnification of NCB and PE-treated CBG from the same donor. A similar structure is visible at lower magnifications illustrating the trabecular pore network both in NCB and PE-treated CBG (Figures 6(a) and 6(b)). At higher magnification SEM micrographs of PE-treated CBG show an exposed collagen fiber network (Figures 6(f) and 6(h)) compared to untreated NCB with the collagen fibers being associated more with bone minerals (Figures 6(e) and 6(g)). The degree of mineralization of collagen fibers appears decreased by PE-treatment but no structural damages could be detected at the level of the hydroxyapatite-collagen matrix.

## 4. Discussion

For enhancement of bone graft transplantation safety and reduction of pathogen transmission various physical and chemical disinfection methods are available including chemical disinfection, thermal treatment, beta or gamma irradiation, and treatment with hydrostatic pressure [11, 12, 14, 16–18, 25, 30–32]. Some disinfection methods, for example, gamma irradiation, chemical disinfection, microwave, or autoclaving have been shown to more efficiently inactivate viruses and bacteria than other treatments [19–23, 33–35], for example, using high hydrostatic pressure or thermal disinfection at 82.5°C with a limited effect on the viability of pathogens while preserving the biomechanical integrity [13, 18]. Thermal disinfection at 82.5°C for at least 15 min was showed to be very efficient to eliminate virus, vegetative bacteria, fungi, and fungal spores, but heat-resistant spores of B. subtilis and C. sporogenes were reduced only by one to two orders of magnitude [13]. Since physical and chemical disinfection procedures may change the biomechanical properties of allograft bone significantly [19, 21, 36–38] the characterization of the allograft's biomechanical performance is of clinical importance [39].

The application of physical disinfection methods may affect both the biological and mechanical properties of bone allografts. Gamma irradiation is commonly used to disinfect bone grafts whereas a standard minimal dose of 25 kGy is recommended by the International Atomic Energy Agency, IAEA [40]. Endres and Kratz described negative biological effects of gamma irradiation (25 kGy) resulting in a maximum immune response of human bone marrow cells on gamma-irradiated bone grafts [41]. Using fluorescence-activated cell sorting (FACS), they observed a distinct shift with excessive cell proliferation of suppressor and cytotoxic T cells, T helper cells and natural killer cells, while the proportion of mature T and B cells was substantially reduced compared to controls without irradiation. Furthermore it was demonstrated that free radical-based damage caused by gamma irradiation is an important pathway of breakdown by cleaving the collagen backbone of bone allografts [19]. In addition, Singhal and coauthors described that the residual elastic strains in the hydroxyapatite phase decrease markedly with increased X-ray irradiation of cortical bovine bone, indicating damage at the hydroxyapatite-collagen interface [42].

Mechanical properties and biomechanical responses of bone allografts can be altered depending on the disinfection method used [43]. According to these findings, significant differences in failure stress and elastic modulus, compared to control samples, were found for gamma-irradiated human cancellous bone specimens with 60 kGy [21]. At a dose of 30 kGy no differences were observed between irradiated human cancellous bone and control [21, 44]. Knaepler and coworkers found that irradiation of porcine cancellous bone with 10 kGy did not impair the stability, whereas a dose of 25 kGy led to a reduction of stability to approximately 65% in uniaxial compression [37]. The same group investigated the biomechanical effects after thermal treatment where 60°C and 80°C showed no effect on compressive modulus, yield point, energy absorption, and maximum stress while treatment with 100°C and autoclaving at 120°C reduced all parameters to 60% and 13% to 25%, respectively, compared to the control group. Using cortical human bone allografts, however, Mikhael and coauthors found no alteration in the biomechanical properties by chemical disinfection alone, chemical treatment and terminal disinfection by gamma irradiation, and chemical disinfection and lyophilization [45]. Kemper and coworkers observed also no effect on the biomechanical properties testing cortical bovine bone treated with a low temperature chemical disinfection process with alternating cycles of vacuum and pressure [46] compared to untreated specimens [47]. The use of ethylene oxide for chemical disinfection has been effectively questioned regarding osteoinductive properties by Munting and coworkers [24]. The authors stated that the ethylene oxide destroyed almost all the bone-inductive capacity. Zhang and colleagues found out that the problem is not within the chemical disinfection with ethylene oxide but into the temperature of the degassing cycle [48]. Exposure to ethylene oxide at 55°C caused an almost complete loss of osteoinductivity whereas the temperature of 40°C resulted in only a slight alteration of the osteoinductivity of demineralized bone powder packed in a gelatin capsule and implanted in Wistar rats. Aspenberg and coworkers found a dose-dependent inhibition of bone induction properties after chemical disinfection with ethylene oxide of demineralized rat femur [22].

Wildemann and coauthors observed the preservation of several native growth factors, for example, bone morphogenetic protein (BMP-2), in human bone allografts after PE disinfection thereby proving the partial maintenance of the graft's osteoinductivity [49]. In addition, PE treatment on nonosseous musculoskeletal tissues, like tendon skin, and cartilage had no influence on collagenous proteins compared to untreated controls [50]. Haimi et al. reported that the chemical disinfection of cortical bone with PE did not influence the biomechanical properties of grafts significantly [28]. The aim of the current study was to investigate the effects of peracetic acid-ethanol treatment on the biomechanical properties of human CBG using uniaxial compression, which, to the authors' best knowledge, has not been reported in the literature before.

To characterize the biomechanical properties of CBG various biomechanical tests, including tension, compression, bending, shear, and torsion, are available [51]. In the present study, we have used axial compression test being a validated and well-accepted method for the biomechanical characterization of cancellous bone [52–59].

The levels of compressive strength (5.9 ± 0.7 MPa versus 2.7 ± 0.4 MPa, *P* < 0.0001), stiffness (Young's modulus) (311.4 ± 34.7 MPa versus 126.5 ± 23.1 MPa, *P* < 0.0001), and fracture energy (140.1 ± 15.3 N mm versus 89.7 ± 15.1 N mm, *P* < 0.001) were significantly decreased upon PE treatment by 54%, 59%, and 36%, respectively. PE-treated CBG demonstrated a 42% increase in ultimate strain (2.6 ± 0.2% versus 3.7 ± 0.5%, *P* < 0.001) and a 51% decrease in apparent density compared to NCB (1.05 ± 0.16 g cm^−3^ versus 0.51 ± 0.18 g cm^−3^).

Corresponding to our results Vastel and coauthors reported that the chemical processing of human cancellous bone with 6 M urea resulted in a 30% reduction of stress and deformation to failure compared to untreated samples [38]. In contrast, other studies demonstrated no influence on mechanical properties after chemical disinfection [45, 47]. However, it is important to point out that the authors in those studies investigated the influence of treatment on cortical bone. Thus, a clear distinction must be made between the effect of chemical disinfection of cancellous as compared to cortical bone differing with respect to architecture, density, and biomechanical performance. While compact bone strengths ranges from 106 MPa to 133 MPa, cancellous bone strength varies between 5 MPa and 10 MPa for axial compression [60]. The compressive properties of NCB in the range of 5.9 ± 0.7 MPa obtained in the present study are in line with a previous report on comparable values of 5.3 ± 2.9 MPa for untreated human cancellous bone from the proximal tibia [61]. The same authors reported a Young's modulus of 445 ± 237 MPa for fresh-frozen CBG which is in the range of our findings with 311.4 ± 34.7 MPa for the untreated specimen. Treated CBGs, often applied as morselized allograft are used to fill bone cavities while cortical bone grafts are applied for the reconstruction of smaller cortical bone defects and for structural support [6, 7]. From this point of view proper biomechanical performance of a cortical bone graft may be of higher clinical relevance compared to that of a CBG whereas the preservation of osteoinductive properties/biocompatibility and favorable remodeling rate are more relevant for CBGs. As in the present study PE treatment reduced compressive strength; it can be argued that the impact on clinical application when used as a filling material without needing structural support in, for example, total joint revision surgery or tumor resection is of minor importance. The clinical use of PE treated cancellous grafts in weight bearing situations, for example, filling of lower extremity critical bone, has to be considered carefully. When seeding with human mesenchymal stromal cells no negative effect in biocompatibility was found after PE treatment as already shown in our previous work [27]. In contrast to the chemical treatment with PE it has been reported that residual ethylene oxide in allografts caused moderate inflammation from residual ethylene oxide and impaired the new bone [7, 62]. Therefore the disinfection with PE can be more recommended for bone graft sterilization when used as a filling material.

Ashby stated that the most important factor affecting the mechanical properties of a porous structure is the relative density [63]. Carter and Hayes suggested that the compressive strength of bone over a very wide range of apparent densities is approximately proportional to the square of its apparent density [53]. Besides that, Ashman et al. demonstrated a relationships between Young's moduli and apparent density for the cancellous portion of the proximal human tibia [64]. In addition, Galante and coworkers found a positive correlation between apparent density and compressive strength of human vertebral bone [29]. In agreement to these results we found a strong positive correlation between compressive strength and apparent density of both NCB and PE-treated specimens (*P* < 0.0001; *r* = 0.75).

Considering the results of the study of Cornu and colleagues where femoral heads that had undergone lipid extraction and experienced reductions of 18.9% and 20.2% in ultimate strength and stiffness, respectively, it can be argued that PE treatment may have an additive effect in our experiment but the defatting step itself reduced the strength and Young's modulus [23]. Carter and Hayes investigated the influence of bone marrow and found that the presence of bone marrow increased the strength, modulus, and energy absorption of specimens at a strain rate of 10.0 per second compared to specimen without bone marrow [53]. These results may support our findings that the bone marrow present in the NCB increases the strength and stiffness and therefor PE treatment itself is not the only parameter responsible for the alteration of the biomechanical properties of CBG.

Dux et al. reported that CBG was damaged by gamma radiation [36]. Using histological evaluation we did not observe any negative effects on the quality of CBG by PE treatment. SEM micrographs of PE-treated CBG demonstrated an exposed collagen fiber network, whereas in untreated NCB the collagen fibers were associated with a higher density of bone minerals. The degree of mineralization of collagen fibers appears decreased by PE disinfection which can explain the increased elastic properties of PE-treated CBG. The observed reduction of stiffness of the PE-treated CBG can be advantageous for implant stability as described by Kold et al. who reported a so-called spring-back effect of compacted cancellous bone reducing the initial gaps between the implant and the cancellous bone bed [65]. PE treatment is likely to increase this spring-back effect thereby supporting implant stability. In addition, Putzier et al. compared PE-treated CBG with autologous iliac crest cancellous bone for lumbar segmental spondylodesis indicating an excellent clinical performance of PE-treated CBG [66]. PE treatment can be regarded as an advantageous disinfection method for bone grafts and potentially for engineered mineralized composite scaffolds paving their way to clinical application.

## 5. Conclusion

In summary, we found that PE treatment reduced compression strength and fracture energy of CBG. However, the elastic properties, as assessed by Young's modulus and ultimate strain, were improved in PE-treated CBG—the latter may lead to a higher deformation reserve of the graft compared to the host bone. SEM revealed a modified microstructure of CBG with exposed collagen fibers after PE treatment. We conclude that the observed reduced compressive strength and stiffness are beneficial during tissue remodeling thereby explaining the excellent clinical performance of PE-treated CBG as a structural graft for localized bone reconstruction. PE-treated CBG can be considered as an appealing matrix for cell-based site-specific bone regeneration and PE-treatment may as well be an attractive disinfection method for other types of porous mineralized composite scaffolds engineered for regenerative therapy of hard tissue.

## Figures and Tables

**Table 1:** Diameter, length, mass, and density of native cancellous bone (NCB) and peracetic acid-ethanol (PE) treated cancellous bone graft (CBA) cylinders (mean ± standard error).

	Diameter (mm)	Length (mm)	Mass (g)	Apparent density (g/cm^3^)
NCB	14.76 ± 0.25	12.13 ± 0.07	1.79 ± 0.28	1.05 ± 0.16
PE-treated CBA	14.36 ± 0.07	12.00 ± 0.06	0.82 ± 0.28	0.51 ± 0.18

**Table 2:** Biomechanical donor-dependent data. Compressive strength (*σ*), Young's modulus (*E*), ultimate strain (*ε*) and fracture energy (FE) of native cancellous bone (NCB) and peracetic acid-ethanol treated cancellous bone graft (PE-treated CBA) cylinders from 4 donors (mean ± standard error; *n* = 6 per donor; m: male, f: female).

	*σ* (MPa)	*σ* (MPa)	*E* (MPa)	*E* (MPa)	*ε* (%)	*ε* (%)	FE (N mm)	FE (N mm)
	NCB	PE	NCB	PE	NCB	PE	NCB	PE
Donor 1 (m)	8.6 ± 1.2	3.0 ± 1.4	473.2 ± 67.2	157.8 ± 91.8	2.4 ± 0.1	3.0 ± 0.4	205.2 ± 29.0	81.0 ± 29.6
	*≙*100%	*≙*35%	*≙*100%	*≙*33%	*≙*100%	*≙*125%	*≙*100%	*≙*39%
Donor 2 (m)	8.2 ± 1.3	2.3 ± 0.6	405.7 ± 50.5	90.0 ± 21.4	2.7 ± 0.3	5.5 ± 1.7	174.6 ± 35.7	114.6 ± 45.9
	*≙*100%	*≙*28%	*≙*100%	*≙*22%	*≙*100%	*≙*204%	*≙*100%	*≙*66%
Donor 3 (m)	2.7 ± 0.5	3.0 ± 0.9	132.3 ± 33.2	126.9 ± 41.3	3.1 ± 0.6	3.5 ± 0.2	76.9 ± 12.3	98.5 ± 24.8
	*≙*100%	*≙*111%	*≙*100%	*≙*96%	*≙*100%	*≙*113%	*≙*100%	*≙*128%
Donor 4 (f)	4.3 ± 0.2	2.4 ± 0.6	234.5 ± 19.3	136.7 ± 34.6	2.4 ± 0.1	2.7 ± 0.2	109.6 ± 6.9	63.1 ± 15.0
	*≙*100%	*≙*56%	*≙*100%	*≙*58%	*≙*100%	*≙*113%	*≙*100%	*≙*58%
Group mean	5.9 ± 0.7	2.7 ± 0.4	311.4 ± 34.7	126.5 ± 23.6	2.6 ± 0.2	3.7 ± 0.5	140.1 ± 15.3	89.7 ± 15.1
	*≙ * **100%**	*≙ * **46%**	*≙ * **100%**	*≙ * **41%**	*≙ * **100%**	*≙ * **142%**	*≙ * **100%**	*≙ * **64%**
